# Soil Microbiome Influences on Seedling Establishment and Growth of *Prosopis chilensis* and *Prosopis tamarugo* from Northern Chile

**DOI:** 10.3390/plants11202717

**Published:** 2022-10-14

**Authors:** David Castro, Christopher Concha, Fabiola Jamett, Cristian Ibáñez, Vaughan Hurry

**Affiliations:** 1 Umeå Plant Science Centre, Department of Forest Genetics and Plant Physiology, Swedish University of Agricultural Sciences, 90736 Umeå, Sweden; 2 Laboratorio de Silvogenómica y Biotecnología, Departamento de Biología, Facultad de Ciencias, Universidad de La Serena, La Serena 1720236, Chile; 3 Laboratorio de Fitoquímica y Productos Naturales, Departamento de Química, Facultad de Ciencias, Universidad de La Serena, La Serena 1720236, Chile

**Keywords:** northern Chile, *Prosopis chilensis*, *Prosopis tamarugo*, Atacama Desert, soil microbiome, plant–microbe interactions

## Abstract

*Prosopis chilensis* and *Prosopis tamarugo*, two woody legumes adapted to the arid regions of Chile, have a declining distribution due to the lack of new seedling establishment. This study investigated the potential of both species to establish in soil collected from four locations in Chile, within and outside the species distribution, and to assess the role of the root-colonizing microbiome in seedling establishment and growth. Seedling survival, height, and water potential were measured to assess establishment success and growth. 16S and ITS2 amplicon sequencing was used to characterize the composition of microbial communities from the different soils and to assess the ability of both *Prosopis* species to recruit bacteria and fungi from the different soils. Both species were established on three of the four soils. *P. tamarugo* seedlings showed significantly higher survival in foreign soils and maintained significantly higher water potential in Mediterranean soils. Amplicon sequencing showed that the four soils harbored distinct microbial communities. Root-associated microbial composition indicated that *P. chilensis* preferentially recruited mycorrhizal fungal partners while *P. tamarugo* recruited abundant bacteria with known salt-protective functions. Our results suggest that a combination of edaphic properties and microbial soil legacy are potential factors mediating the *Prosopis* establishment success in different soils.

## 1. Introduction

Chile is a country that extends from 17°30′ to 56°30′ southern latitude, encompassing highly variable climates ranging from class B (dry climate) to class E (polar and alpine climate) in the Köpper–Geiger climate classification [1]. Northern Chile, which extends from 17°30′ to 32°16′ southern latitude and spans the Arica and Parinacota, and Coquimbo regions, contains the arid and semi-arid zones of the country characterized by high temperatures and low precipitation [2,3]. The Atacama Desert, considered one of the oldest and driest nonpolar deserts in the world, is located between 19° and 30° southern latitude and extends approximately 1000 km from north to south [3,4]. The Atacama Desert’s extreme conditions of dryness, salinity and toxic metal concentrations make these soils hostile for most living species [5]. It has been proposed that the Atacama Desert will expand further south in response to reductions in precipitation and increases in temperature driven by climate change [6].

In order to survive in environments as harsh as arid and semi-arid zones some organisms, known as extremophiles or extremotolerant, have evolved to survive one or more extreme conditions, for example, high salt or toxic metal concentration, high radiation, water deficit, or desiccation [4,5,7,8,9,10,11]. While extremophiles, particularly prokaryotes, have developed different metabolic pathways, such as chemolithoautotrophy, enabling them to grow in hostile soil environments [10,12,13]; non-extremophiles have developed alternative strategies, including mechanisms to escape (e.g., CAM photosynthesis [14]), or tolerate abiotic stress (e.g., osmolyte accumulation in the cells [15]), as well as establish mutualistic interactions (e.g., microbial partners with ACC deaminase activity [16], or that modulate hormone and antioxidant activity [17]) to help them cope with environmental stress [18,19,20]. For instance, several studies have found that plants establish interactions with soil microbiomes, including archaea, bacteria, and fungi [21] that have beneficial effects on growth during drought [22], and salt stress [23], high or low nitrogen content [24], pathogen presence or heavy metal content [25], by modulating phytohormones levels or producing protective compounds.

The dominant plant group in the arid region of Chile is from the genus *Prosopis*, the members of which are woody leguminous shrubs or trees that are tolerant to extreme salt and drought stress [26]. *Prosopis* species have documented interactions with both arbuscular and ectomycorrhizal symbionts [27,28,29], as well as bacteria from the genus *Rhizobium* [28,30]. Eight species of *Prosopis* have been described from Chile, distributed from 17° to 35° south latitude, with most concentrated in the north in the Tarapacá and Atacama regions (Figure S1) usually along old dry river courses [31]. Every year *Prosopis* spp. produce abundant pods, rich in sugars and protein, that are traditionally used for livestock feed and to make flour, while the timber has been used as fuel [32,33,34,35]. Heavy usage of these species has reduced their historical distribution, leading to six of the species to be listed as vulnerable, endangered, or critically endangered (Figure S1). The combination of climate change-induced increased desertification [6] and the current lack of recruitment of new seedlings [36] has increased the risk of local extinction of some *Prosopis* spp. [34,36,37].

Seeds from *Prosopis* species have a hard coat that weakens through time, although passage through a digestive tract is known to clean the seed coat and break seed dormancy [35]. Previous studies have demonstrated the capacity of *Prosopis* seeds to germinate under NaCl concentrations up to 600 mM [34,38], suggesting that *Prosopis* species can establish in relatively high saline soils. Similarly, Sohrabi and Gherekhloo (2012) [39] and Aljasmi et al. [40] found that *Prosopis* species could germinate under osmotic stress equivalent to −1.0 MPa, suggesting that *Prosopis* species could also establish in dry environments. In addition to the capacity of the seeds to germinate in adverse conditions, it has been shown that *Prosopis* seedlings can successfully establish and grow in saline substrates [41] and tolerate long periods of water deprivation [31], suggesting that factors other than abiotic stress could be impairing seedling establishment success. One of the most well-known characteristics of *Prosopis* spp. is its fast-growing, long taproot system that allows it to reach deep water sources and a shallow secondary root system that allows it to uptake water from more superficial soils [35], indicative of the importance of a well-developed root system for establishment success. Due to its ability to lift water from deep layers and to act as a nursery plant, the genus *Prosopis* has been suggested as a good candidate for reforestation, soil stabilization and slowing the desertification processes [34,42,43]. In addition, *Prosopis* spp. have the ability to grow in the presence of, and sequester heavy metals, making them potential candidates for phytoremediation [44,45]. The high tolerance of *Prosopis* spp. has made them aggressive invaders in other arid and semi-arid regions, such as in southern Africa and Australia, causing great ecological damage [46]. However, the question of why *Prosopis* spp. Are close to local extinction in Chile remains unanswered. Here, we assess the potential of two *Prosopis* species (*P. chilensis* and *P. tamarugo*) from different locations to establish and grow in soils from four different places of Chile (i.e., within (native) and outside (foreign) the genus natural distribution) with very different edaphic properties. Additionally, we assess the ability of these two species to recruit a root-associated microbiome from these different soils. Using a factorial design of soil types-*Prosopis* spp., we aim to provide new insight to help explain the lack of seedling recruitment within the genus’ natural distribution.

## 2. Results

### 2.1. Soil Characterization

**Figure 1:**
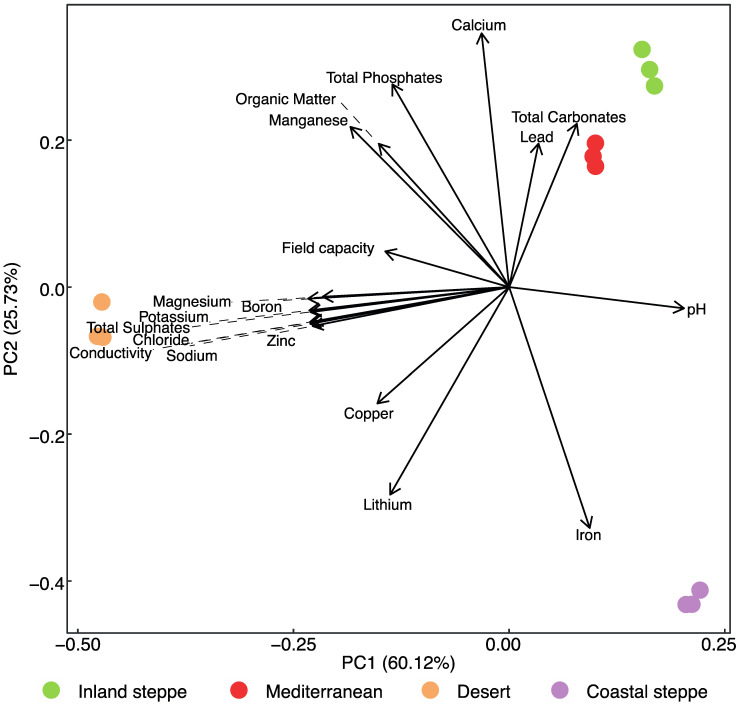
Principal component analysis (PCA) biplot depicting the different soils and edaphic properties. For graphical purposes, dashed lines were added to extend the vectors to connect to the corresponding labels when needed. Colored by location from Figure S1.

The mineral content of the four soils differed significantly (PerMANOVA *p*-value < 0.001), with the Desert and Coastal steppe soils being the most divergent (Figure 1). Desert soil (Pozo Almonte) had extremely high contents of Potassium (K^+^), Boron (B^−^), Chloride (Cl^−^), Sodium (Na^+^) and Total Sulphates (SO_4_^2−^), which were the factors that drove the divergence through component one (Table 1; Figure 1). The factors that drove the separation through the second component included Calcium (Ca^2+^), Iron (Fe), Lithium (Li), Total Phosphates (PO_4_^3−^) and Total Carbonates (CO^2−^). From these, Ca^2+^ was higher in both Mediterranean (Los Angeles) and Inland steppe soils (Vicuña) (Table 1). Coastal steppe soil (La Serena) had a high Fe content (Table 1), which may be a consequence of its proximity to an iron ore deposit that connects to the site through a dry riverbed where mine tailing residues could be carried to the site through runoff during the rainy season. Inland steppe soil had high concentrations of Lead (Pb), Li and an alkaline pH and low organic matter, as well as high concentrations of PO_4_^3−^ and SO_4_^2−^ (Table 1). Mediterranean soil, on the other hand, had low conductivity and an acidic pH, together with high organic matter (Table 1). The field capacity of the soils suggests that both Desert and Inland steppe soil had better water holding capacity compared to Coastal steppe and Mediterranean soils (Figure S2).

### 2.2. Seedling Establishment and Growth

**Figure 2:**
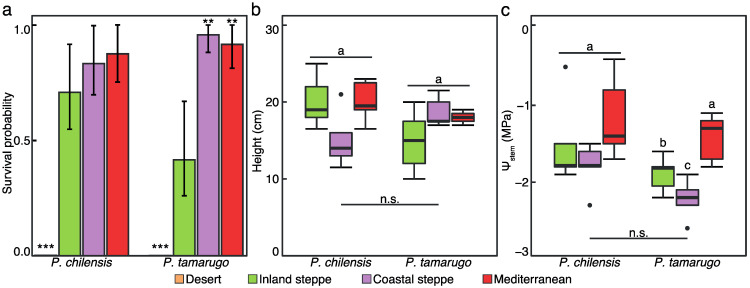
Bar plot of the survival probability (**a**) and boxplots of height (**b**) and stem water potential (**c**) of *P. chilensis* and *P. tamarugo* in different soils, colored by location. In (**a**) asterisks (** *p* < 0.01, *** *p* < 0.001) represent statistical differences of Cox proportional Hazard regression for *n* = 24. Error bars correspond to 95% confidence interval. In (**b**,**c**) lowercase letters in the upper part of the boxplot represent Fisher’s least significant difference (LSD) when α = 0.05 from Kruskal–Wallis testing differences between soils. Whiskers represent 1.5× inter-quartile range (IQR). Dots correspond to outliers. Lowercase letters below the boxplots correspond to statistics from Kruskal–Wallis testing differences between *Prosopis* species.

The four different soils selected for this study covered the native distribution for both *Prosopis* species. The Atacama Desert soil was native for *P. tamarugo* and the Inland steppe was native for *P. chilensis*. In addition, two soils considered foreign for both species were used: Coastal steppe, located within the historical native range of both species but not currently colonized by either species and Mediterranean soil located outside the native range (Figure S1). After two months of growth, both species showed significant differences in survival depending on soil type. All seedlings of both species died within a few days of being sown in Desert soil (Figure 2a). Seedlings were re-sown twice, but no seedlings were able to establish in this soil type (Data not shown). When comparing the other three soil types, no overall differences in survival were found between *P. chilensis* and *P. tamarugo*. However, both species showed a trend for lower survival in Inland steppe soil, and this reduced survival was significant for *P. tamarugo* (Figure 2a). Further, both *Prosopis* species showed no overall differences in stem water potentials and both species tended to maintain higher water potentials in Mediterranean soils, although this was only significant for *P. tamarugo* (Figure 2c; *p*-value < 0.05). Once established, both *Prosopis* species showed similar height growth irrespective of soil type (Figure 2b). Thus, the lower water potentials maintained by seedlings in Coastal and Inland steppe soils, especially for *P. tamarugo*, did not appear to limit the growth of the seedlings once established, assessed by height growth, or to be the differentiating factor behind lower seedling survival of this species, suggesting that there were additional factors contributing to lower seedling recruitment in soil from this region.

### 2.3. Soil Microbial Community

**Figure 3:**
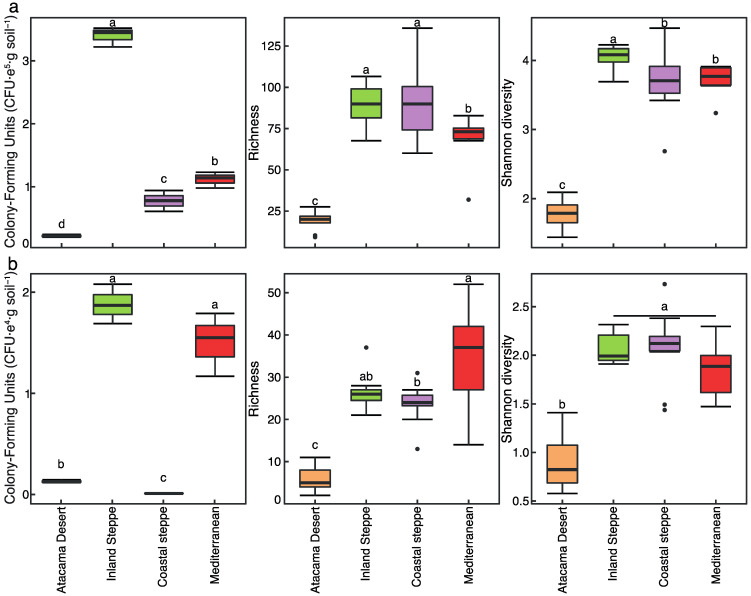
Boxplot of bacterial (**a**) and fungal (**b**) Colony-Forming units, Richness and Shannon diversity index of soil samples, colored by location. Lowercase letters in the upper part of the boxplot represent Fisher’s least significant difference (LSD) when α = 0.05 from Kruskal–Wallis testing differences between soils. Whiskers represent 1.5× inter-quartile range (IQR). Dots correspond to outliers.

Microbial load in the different soils was assessed by first estimating colony-forming units (CFUs). From this, Inland steppe soil had the highest bacterial load of all soils (*p*-value < 0.05), being about three times higher than Mediterranean soil, the second highest. Desert soil had the lowest bacterial load, being about four times lower than Inland steppe soil (Figure 3a). Similar to bacterial CFUs, fungal CFUs showed the highest load in the Inland steppe together with Mediterranean soils (*p*-value < 0.05). Coastal steppe soil had the lowest fungal CFUs, representing about 106 CFUs per gram of soil, being 10 times lower than Desert soil with about 1290 CFUs per gram of soil (Figure 3b).

The sequenced amplicon data successfully annotated 956 bacterial amplicon sequence variants (ASVs) across all samples. These ASVs were used to assess the richness and diversity of the bacterial community (Figure 3a) in the different soil samples. The Desert soil had the lowest values of bacterial richness and diversity (Figure 3a; *p*-value < 0.05). Inland steppe and Coastal steppe soils, on the other hand, shared the highest bacterial richness values. However, Inland steppe had higher Shannon diversity values (*p*-value < 0.05), indicating that in the Inland steppe soil the bacterial community was more diverse, with fewer dominant bacteria, than in the Coastal steppe soil (Figure 3a). From the sequenced fungal reads, 262 ASVs were successfully annotated and used to estimate the richness and diversity of the fungal community (Figure 3b). Similar to the bacterial community estimates, Desert soils had the lowest values of richness and diversity (Figure 3b; *p*-value < 0.05). Mediterranean soil had the highest fungal richness value, followed by Inland steppe. Despite large differences in richness, no statistical differences were found in diversity between Inland steppe, Coastal steppe, and Mediterranean soils (Figure 3b).

**Figure 4:**
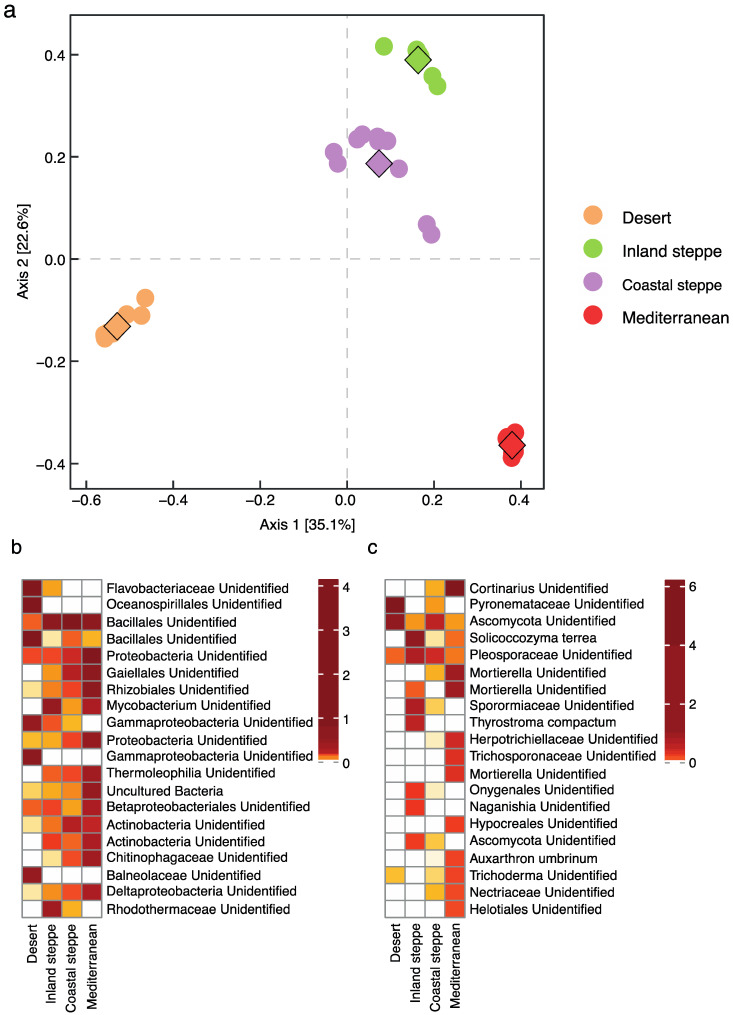
Principal coordinate analysis (PCoA) of the microbial community composition of soil samples (**a**). Diamonds correspond to the group centroid. Heatmaps showing the top 20 bacterial (**b**) and fungal (**c**) ASVs that drove the community differences. Color scale was grouped by quintiles for visualization purposes. See Table S4 for dissimilarity analysis and Table S5 for full microbial community.

In addition to microbial community estimations, ASVs were used to assess the composition of the bacterial and fungal communities. Principal coordinate analysis (PCoA) suggests that the composition of the bacterial communities from all four soils differed significantly (PerMANOVA *p*-value < 0.001), but the two geographically close steppe soils were the most similar (Figure 4a). The composition of the bacterial community in Desert soil was the most different, and dissimilarity analysis indicated that seven ASVs were the most dissimilar. From these seven ASVs, four annotated as Flavobacteriaceae, Oceanospirillales, Bacillales and Gammaproteobacteria were the ones that made the Desert soil community the most distinct from the others (Figure 4b; Table S4). Interestingly, members of all these taxa are known to have adaptations to high-salinity environments [52]. Along axis two, Mediterranean soil separated from the two steppe soils (Figure 4a). From dissimilarity analysis, 13 ASVs were found to drive this difference, and ASVs annotated as Proteobacteria, Gaiellales, Rhizobiales, Thermoleophillia, Chitinophagaceae and Deltaproteobacteria were the most abundant in Mediterranean soil (Table S4). The presence of these taxa suggests that the Mediterranean soil is rich in fungal necromass and plant-derived carbon sources, especially chitin (Chitinophagaceae) [53] and N-alkanes (Thermoleophillia) [54,55] (Table S4). In addition, the high abundance of Rhizobiales suggests that the Mediterranean soil hosts a bacterial community beneficial for plant establishment [56,57].

Similar to the bacterial communities, PCoA of the fungal communities suggests that all soil types harbor different fungal communities (PerMANOVA *p*-value < 0.001), with axis one separating the fungal communities from northern (Desert, Inland and Coastal steppes) and southern soils (Mediterranean). Dissimilarity percentage analysis indicated that six fungal ASVs differentiate the Mediterranean soil, with ASVs annotated as *Cortinarius*, a genus containing well-known ectomycorrhizal species, being the most abundant (Figure 4c). Along axis two, eight fungal ASVs were responsible for separating the Inland steppe soil from the other sites, with ASVs annotated as *Solicoccozyma terrea*, a yeast known to produce and deliver indole-3-acetic acid (IAA) to the plants and to have potential as a pathogen antagonist [58], being the most abundant (Figure 4c). Coastal steppe soils contained 10 ASVs that drove the differences from the other soils, with one of these being annotated as Pleosporaceae, which corresponds to a family with a large number of saprotrophic fungi [59] (Table S4).

### 2.4. Root-Associated Microbial Community

After two months of growth in the different soils, the root-associated microbiome of both *Prosopis* species was assessed using the bacterial and fungal ASVs obtained from amplicon sequencing. As all seedlings sown in the Desert soil died, this soil type was removed from this analysis. Thus, for this section, soils will be referred to based on the natural distribution of the *Prosopis* spp. (Figure S1). Accordingly, Inland steppe soil will be referred to as native soil within the genus distribution (native-within; NW), Coastal steppe as foreign soil within the genus distribution (foreign-within; FW) and Mediterranean soil as foreign soil outside the genus distribution (foreign-outside; FO).

**Figure 5:**
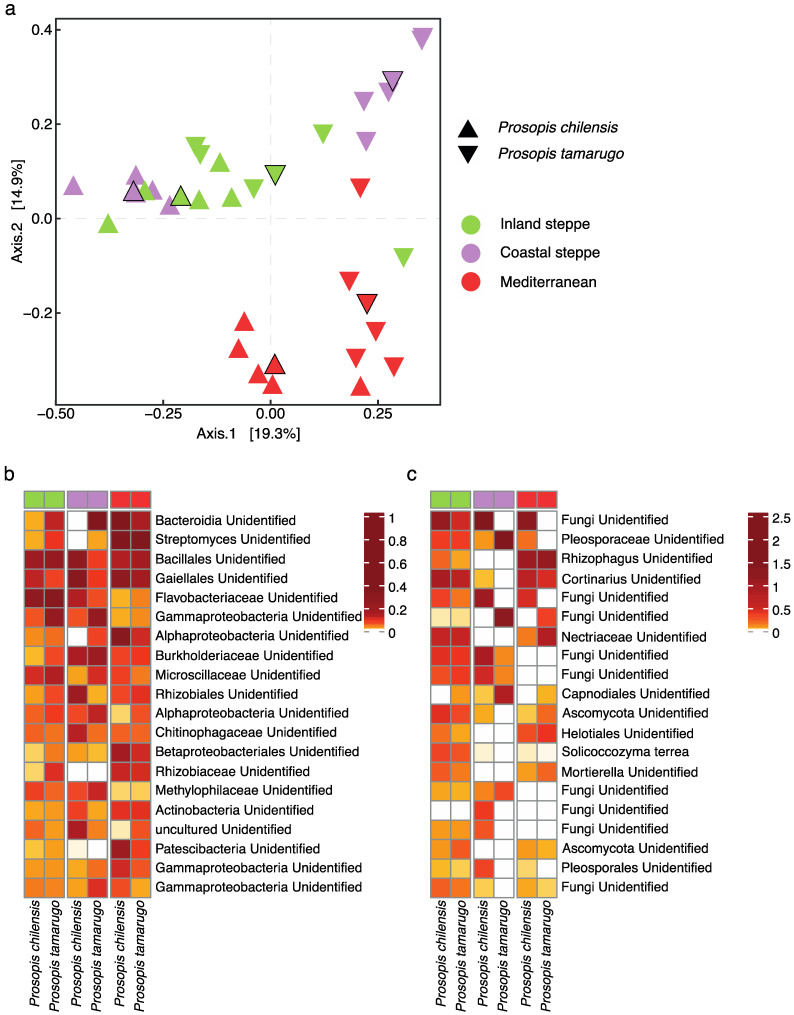
Principal coordinate analysis (PCoA) of the microbial community composition of soil samples (**a**). Black bordered shapes correspond to the group centroid. Heatmaps showing the top 20 bacterial (**b**) and fungal (**c**) ASVs that drove the community differences. Color scale was grouped by quintiles for visualization purposes. See Tables S6 and S7 for dissimilarity analysis.

To assess the differences between the bulk pot soil community and the recruited microbiome, the relative abundance of the bulk pot soil microbiome was contrasted with the root-associated microbiome (Table 2). Both *Prosopis* species showed similar proportions of root-associated bacterial ASVs in NW, reaching about 36% in the whole community, explaining the high compositional similarity of the root-associated bacterial communities of the two *Prosopis* species observed in this soil (Table 2; Figure 5a). Within the common bacterial ASVs, *Rhizobia*, *Bacillus*, *Streptomyces*, Cytophagales and Burkholderiaceae were among the most abundant in the root-associated communities of both *Prosopis* species (Figure 5b). Notably, Cytophagales is a bacterial order that is known to contain important mineralizers [60], while Burkholderiaceae is a bacterial family that contains members with nitrogen (N) fixation and phosphorus mineralization functions [61,62], suggesting that the roots of both *Prosopis* species were colonized by bacterial partners with potential beneficial functions, with *P. tamarugo* having a greater relative abundance of these potentially beneficial partners (Figure S3a). *Prosopis chilensis* showed reduced bacterial root colonization in both foreign soils, accounting for only 11% of bacterial ASVs enriched in the roots in both FW and FO soil, while *P. tamarugo* accounted for 28 and 37% of bacterial ASVs in FW and FO soils, respectively (Table 2). In addition to this change in root assembly behavior, there was a lower overlap in the root-associated bacterial communities (Figure S3a—Coastal steppe). Within the bacteria from FW soils found on the roots of both *Prosopis* species, Bacillales, Burkholderiaceae, Cytophagales and *Rhizobia* were the most abundant (Figure 5b). Finally, in FO soils *Streptomyces*, Bacillales and Burkholderiaceae were most abundant on the roots of both *Prosopis* species, with *Streptomyces* and Burkholderiaceae being more abundant on *P. tamarugo* (Figure 5b).

Similar to what was observed for the bacterial community, the root-associated fungi had the highest values of fungal ASVs enriched in the roots in NW soil, accounting for 38 and 29% for *P. chilensis* and *P. tamarugo*, respectively (Table 2). These ASVs were dominated by *Rhizophagus* and *Cortinarius*, arbuscular [63] and ectomycorrhizal fungi, respectively, and *Solicoccozyma terrea*, a yeast that is known to produce IAA and deliver it to the plant roots. While these three fungi were dominant on the roots of both *Prosopis* species in NW soil, they were all more abundant on *P. chilensis* (Figure 5c). Both *Prosopis* species had the lowest root colonization of fungal ASVs when sown in FW soil, with *P. chilensis* recruiting only 11% and *P. tamarugo* 5% of the total community, respectively (Table 2). In FO soil, mycorrhizal fungi colonization was greater than observed in FW soil (Table 2), with both *Prosopis* species showing high relative abundance for *Rhizophagus* and *Cortinarius* in FO soil (Figure 5c).

Analysis of the unique root-associated bacterial ASVs in both *Prosopis* species growing in NW soil showed that while both species had a high number of unique bacterial ASVs, both species assembled functionally similar bacterial partners, with mineralizers, such as members of Bacteroidia, N-fixers, such as *Rhizobia*, and osmotic and salt resistant Bacillales being the most abundant (Table S6). In foreign soils, in addition to the low community overlap found in FW and FO soils, *P. tamarugo* had about five to six times more unique bacterial ASVs than *P. chilensis* (Figure S3a), further demonstrating that *P. tamarugo* has greater preference or facility for being colonized by bacterial rather than fungal partners. Within these unique bacterial ASVs, *P. tamarugo* had a high abundance of bacteria annotated as salt-tolerant (e.g., Oceanospirillales, *Haliangium*, *Bacillus*) as well as N-fixing (e.g., *Rhizobia* and *Paenibacillus*) in both foreign soils, although in greater proportion in FW than in FO soil (Table S6). *Prosopis chilensis*, on the other hand, showed little selectivity in root-associated bacterial assembly in FW and FO soils, and the unique ASVs found in the roots were mostly generalist, although a few mineralizers and *Rhizobia* were found (Figure S3a; Table S6).

In contrast to the pattern of community assembly of unique bacterial ASVs, the *P. chilensis* root-associated community accounted for more unique fungal partners in NW soils, having about double the number of fungal ASVs than *P. tamarugo* (Figure S3b). The composition of the unique fungal community suggested that *P. chilensis* assembled a root-associated community with a large number of opportunistic fungi, although with low relative abundance. These include the leaf pathogen *Thyrostroma compactum* [64] and *Arthrobotrys elegans*, which have been described as nematophagous [65,66] (Table S7). *Prosopis tamarugo*, on the other hand, showed a high abundance of pathogenic fungi from the *Erysiphe* genera [67] and the black yeast *Aureobasidium pullulans*, which has been described as having anti-pathogenic functions [68,69]. These results suggest that pathogenic fungi are colonizing the roots of both *Prosopis* species in their native soils (Figure 2a). In FW soils, *P. chilensis* roots were colonized by about double the number of unique ASVs as *P. tamarugo* (Figure S3b), although most of these were annotated as unknown, with a few, such as *Cortinarius*, *S. terrea* and *Fusarium*, successfully annotated (Table S7). Unique fungal ASVs recruited by *Prosopis tamarugo* were dominated by *Debaryomycetaceae* spp., which corresponds to a yeast family often found within insect guts [70] and a decomposer from the Onygenales order [71] (Table S7). Finally, a similar number of unique fungal ASVs were found in FO soils (Figure S3b), with *P. chilensis* being colonized by members of the Fusarium genera, as well as the arbuscular mycorrhiza *Diversispora epigaea* [72], and the beneficial soil yeast *S. terrea* (Figure 5c, Table S7). The unique fungi colonizing *P. tamarugo* roots, on the other hand, were mostly annotated as opportunistic fungi. Altogether, the different patterns of root-associated community assembly of both *Prosopis* species emphasize the greater capacity of *P. chilensis* for fungal recruitment, relative to *P. tamarugo*, both within and outside their native range. However, this enrichment in fungal colonization may also indicate root colonization by commensals or parasites, as suggested by the presence of opportunistic fungi like Helotiales and Capnodiales in the roots of *P. chilensis* in the three soils.

## 3. Discussion

Soil properties and the soil microbiome have major effects on plant establishment, growth, and distribution [73,74]. In the Chilean deserts, *Prosopis* species stand out due to their high tolerance to harsh conditions, but their distributions are narrowing due to a lack of regeneration, with some species close to extinction [36]. Here, we studied the impact of soil properties and the soil microbiome on seedling establishment and growth of two *Prosopis* species, *P. chilensis* with a wide distribution and *P. tamarugo*, with a narrow distribution (Figure S1), to understand the potential factors limiting the establishment of new seedlings in their natural environment. Our results suggest that both *Prosopis* species have the potential to establish in native and foreign soils, including Coastal steppe soils, but seedlings, even of the endemic species *P. tamarugo*, were not able to establish and grow in the extreme conditions of the Atacama Desert soil (Table 1, Figure 2). Soil microbial composition analysis showed that each soil harbors a distinct microbiome, presumably adapted to the specific properties of each site (Figure 4). Both *Prosopis* species recruited larger and more complex root-associated microbiomes from native soil, suggesting that a strong historical relationship between both *Prosopis* species and the soil microbiome is important for recruitment (Table 2, Figure S3). In foreign soils, *P. tamarugo* roots were colonized by a larger number of unique bacterial partners with beneficial properties, such as *Haliangium* and *Paenibacillus*, with salt-tolerance and N-fixation potential, respectively (Table S6), than *P. chilensis*, whose root-associated bacterial community in foreign soils was mostly generalist (Table S6). Fungal community assembly demonstrated that the root system of both *Prosopis* species could be colonized by mycorrhizal partners, such as *Cortinarius* and *Rhizophagus*, with *P. chilensis* being more abundantly colonized by these fungi than *P. tamarugo*, especially in native soil (Figure 5; Tables S6 and S7). Our results suggest that, in addition to soil edaphic factors, the composition of the soil microbial community and the differences between *P. chilensis* and *P. tamarugo* in their root-associated microbial community assembly behavior are important components mediating *Prosopis* seedling establishment.

The evolutionary history of *Prosopis* suggests that the genus has co-expanded with deserts, with an ancient association with xeric zones as early as 33 Mya [26]. In Chile, early *Prosopis* records place *P. tamarugo* in the Atacama Desert about 14,600 calibrated years before the present [75]. Soil microbiomes are shaped by the soil properties and the associated plant communities. This phenomenon, known as soil legacy, has been found to have either positive or negative effects on seedling establishment success, depending on the diversity of plant species already established and the species of the new seedlings established in the soil (see Shen et al. [76] and Zhang et al. [77]), with native species being favored by native plant-soil legacy. Thus, to understand the potential implications of the soil microbial legacy in the lack of recruitment of new *Prosopis* seedlings in Chile, non-native soils within and outside its distribution range (FW and FO) were used together with native soil (NW) in this study. The bacterial and fungal root colonization analyses suggest that both *Prosopis* species have greater microbial receptivity in NW soil, with a higher degree of community similarities in native than in foreign soils (Table 2; Figure 5 and Figure S3), consistent with a historical soil legacy. However, although significantly divergent in both bacterial and fungal composition (Figure 4), both FW and FO soils provided potentially beneficial microbial communities for the two *Prosopis* species used in this study, as judged by a complex root-associated microbiome assembly and seedling survival rates and growth (Figure 2). Nevertheless, species-specific assembly patterns were observed in both FW and FO soils, where *P. chilensis* roots were consistently colonized by more fungal partners while *P. tamarugo* were colonized by more bacterial partners. This suggests that *P. tamarugo*, which has been isolated in the Atacama Desert for many decades, has a reduced capacity for fungal colonization relative to *P. chilensis*. In particular, *P. tamarugo* has a strongly reduced capacity for being colonized by symbiotic mycorrhizal partners, such as *Cortinarius* and *Rhizophagus* relative to *P. chilensis*, even in FO soil that is permissive for the growth of these fungi. Instead, *P. tamarugo* roots were colonized by more beneficial bacterial partners with nutrient mineralization, N-fixation and salt-protection functions. This suggests that the historical relationship of *P. tamarugo* might be toward establishing interactions with bacterial partners with these nutrient mobilization functions, which would be consistent with its persistence in the Atacama Desert and its soil microbial legacy there. Nevertheless, despite the ancient association of *Prosopis* spp. with xeric zones, Inland steppe (NW) soil provided the second least favorable conditions for seedling establishment after Desert soil, with *P. tamarugo* showing significantly lower survival and lower stem water potentials in this soil (Figure 2). One potential limitation for *P. tamarugo* in Inland steppe soil could be the poor colonization of mycorrhizal fungal partners, given their well know ability to supply not only nutrients but also water to their host roots [78]. Similarly, *P. tamarugo* roots were more colonized by *Rhizophagus* in FO soil than NW soil, potentially contributing to the significantly higher water potential in FO soil (Figure 2c and Figure 5c). In addition, the presence of potentially harmful pathogens in the *P. chilensis* and *P. tamarugo* root-associated communities may also provide insight into *Prosopis* seedling establishment. For example, analysis of fungal assembly by *P. chilensis* showed that Helotiales, Mortierella and Capnodiales, all opportunistic and potentially harmful fungi [79], were consistently amongst the most abundant fungi in all soil types, but particularly in FW and FO soils. When taken together, these results suggest that opportunistic fungal load might be a factor in the recruitment of new *Prosopis* seedlings across the distribution range in Chile, but without a detailed analysis of the interaction between *Prosopis* spp. and these different opportunistic fungi, it is not possible to establish the potential ecological role of the high abundance of Helotiales, Mortierella and Capnodiales in these root samples. In a related study assessing the bacterial recruitment of *P. juliflora* in non-native soils, Kaushik et al. [80] found that the rhizosphere microbiome was enriched in bacterial partners, such as *Streptomyces*, *Isoptericola* and *Brevibacterium*, with enhanced functions in antimicrobial biosynthesis and degradation. In their study, the authors suggested that *P. juliflora* might have been under pathogen attack and that the enrichment of bacterial partners with antimicrobial activity was to counter the pathogens [80]. In our results, we also see in *P. tamarugo* roots an enrichment of bacterial recruitment, with a high abundance of beneficial bacteria, such as *Streptomyces* which, together with the presence of opportunistic fungi, might suggest selective bacterial recruitment toward protection against fungal attack, but further studies will be needed to establish the nature of these relationships within the *Prosopis* root microbiome.

*Prosopis* species are well known for their high tolerance to salt and drought stress [35], and due to this, some species, such as *P. juliflora* and *P. chilensis*, have been used as a soil recovery species in a number of countries including India, Yemen and Kenya [35,81]. However, while *Prosopis* species show high growth when used for this purpose, they have quickly become invasive species [46,81]. The high adaptability shown by *Prosopis* species in foreign soils, in addition to their rapid growth and uncontrolled dispersion, contrasts with the distribution decline that has been observed within the natural ranges of the various *Prosopis* species growing in Chile. Our results suggest that a combination of the soil legacy and the historic relationship between *Prosopis* species and the soil microbiome are potential factors mediating the recruitment of new seedlings within the native range. This response was more noticeable in *P. tamarugo*, which recruited more bacteria and showed a reduced capacity to recruit beneficial fungal partners, consistent with its current isolated distribution in the Atacama Desert. Notably, Atacama Desert soils lacked all potentially beneficial fungi found in the other three soils and future work will be needed to determine the composition of the microbial community associated with the roots of adult *Prosopis* spp. trees currently growing in the Atacama Desert to determine the importance of plant-microbiome interactions for plant survival in the field in this environment.

## 4. Materials and Methods

### 4.1. Plant Description and Seed Sampling

To assess the ability of *Prosopis* species to establish and recruit a root-associated microbiome from soils from different Chilean locations we selected two *Prosopis* species, *P. chilensis* ((Mol.) Stunz.) and *P. tamarugo* (Phil.), from two different environments. The first is a native phreatophyte tree naturally distributed in Argentina, Bolivia, Chile and Perú. In Chile, it is present in seven of the eight regions where the genus is distributed, being locally extinct in Antofagasta (Figure S1), growing in Desert, Inland steppe, and high desert and steppe climates (Figure S1). The second, a xerophyte tree endemic to northern Chile, is restricted to the hyper-arid region of the Atacama Desert of Tarapacá and Antofagasta (Figure S1). Both *Prosopis* species have long taproot systems that reach deep water sources, and a shallow secondary root system that is responsible for absorbing water from the surface following rains. In January 2018, pods from *P. tamarugo* and *P. chilensis* were collected in the Pampa del Tamarugal natural reserve (20°28′12″ S, 69°40′22″ W) and Fundo Maitencillo (29°58′38″ S, 70°45′58″ W), respectively (Figure S1). After collecting the pods, they were stored at 4 °C for at least two months before seed collection.

### 4.2. Soil Sampling and Characterization

Chilean soils vary considerably from north to south. Generally speaking, the pH of northern soils is relatively alkaline, ranging from 6.1 to 8.4 [45], they have low organic matter (~5%) and aggregate stability, high bulk density and high water dispersion [45], making these soils vulnerable to erosion. Moving south, organic matter increases (between 5 and 10%) as well as aggregate stability, while bulk density and water dispersion decreases [45]. Finally, Coastal soils are relatively inert, with low bulk density, neutral pH (6.3–7.2) and low electrical conductivity [45]. To determine the establishment and growth of both *Prosopis* species, the soil within the genus natural distribution range (i.e., Desert and Inland steppe), together with soil within the genus natural distribution but not colonized by either *P. chilensis* or *P. tamarugo* (i.e., Coastal steppe) and soils outside the genus natural distribution (i.e., Mediterranean soil) were collected in the Pampa del Tamarugal natural reserve in Pozo Almonte for desert soil (20°28′12″ S, 69°40′22″ W), Quebrada Marqueza in Vicuña for Inland steppe soil (29°51′59″ S, 70°50′41″ W), Punta Teatinos in La Serena for Coastal steppe soil (29°49′29″ S, 71°17′16″ W) and Human in Los Angeles (37°25′56″ S, 72°13′52″ W) for Mediterranean soil (Figure S1). To characterize the soils, one kilogram of each was homogenized by mixing in a sterile plastic-hermetic bag. After homogenization, soil subsamples were submitted to the central laboratory of analytical chemistry of Universidad de La Serena where mineral content, organic matter and conductivity were measured (Table S1). To assess soil field capacity, we used a modified version of the method used by Haas et al. [82]. Briefly, wet soils were weighed daily until constant weight and the differences between the wet soil and dried soil were determined, results are shown as moisture percentage. To assess soil microbial colony-forming units (CFUs), 10 g of soil was resuspended in 90 mL of sterilized distilled water and serial dilutions were made. Each of the diluted samples was cultured in trypticase soy agar (TSA) for bacterial growth and potato dextrose agar supplemented with 100 μg × mL^−1^ of Kanamycin A for fungal growth. CFUs were counted after 24 h at 25 °C for bacteria and four days at 25 °C for fungi.

### 4.3. Greenhouse Experiment

After at least two months of storage at 4 °C, collected pods were considered ready for seed collection, which was made following the method proposed by Westphal et al. [34]. Briefly, pods were air dried at 25 °C until completely dried and seeds were collected and pooled. Healthy seeds were selected and scarified using 95% sulfuric acid for five minutes and then washed with running water. Scarified seeds were put in a petri dish with sterile distilled water and left for germination at 24 °C. After 24 h, radicle protrusion was observed in about 90% of the seeds. Germinated seeds with a radicle of about two centimeters long were considered ready for the experiment. In total, 96 seedlings of each species were selected and sown into one of the four different soils following a factorial design. One seedling was sown into a pot containing around 550 cm^3^ of soil and placed in a greenhouse in Universidad de La Serena and watered daily. After two months, survival, growth and seedling stem water potential (Ψ_stem_) were measured, and roots and bulk pot soil samples were collected. Stem water potential was measured at midday on recently cut stems sealed into a Scholander-type pressure chamber (PMS Instrument Company Model 1000, Albany, OR, USA). Root and bulk pot soil samples were freeze-dried and stored at −80 °C until further use.

### 4.4. Genomic DNA Extraction, PCR Amplification and Sequencing

Genomic DNA, PCR amplification and sequencing were performed following the method from Castro et al. [83]. Briefly, DNA from mortar-ground freeze-dried roots was extracted using a Cetyl TrimethylAmmonium Bromide (CTAB) based method [84]. Co-extracted RNA was eliminated with RNase A (10 mg × mL^−1^). Freeze-dried bulk pot soil samples were further air dried at 30 °C overnight prior to use and DNA was extracted using the Dneasy PowerLyzer PowerSoil Kit (QIAGEN, The Netherlands) following the manufacturer’s instructions. Purity and concentration were assessed using an Eppendorf BioPhotometer D30 (Eppendorf AG, Hamburg, Germany). Before PCR amplification, DNA samples were diluted to a concentration of 5 ng × μL^−1^. A two-step PCR amplification was performed based on the procedure proposed by Beckers et al. [85]. Amplification PCR was conducted with the primers gITS7 and ITS4 (Table S2) targeting for the internal transcribed spacer (ITS) region were used to amplify the fungal ITS2 region [86,87] and primers pair 799F and 1193r (Table S2) were used to amplify region V5, V6 and V7 of the prokaryote 16S rRNA gene [88]. The PCR reactions were performed using SapphireAmp Fast PCR MasterMix (TaKaRa Bio, Kusatsu, Shiga, Japan) in a final volume of 20 μL. The PCR amplification was run in duplicate for each sample. Five min at 95 °C was used for initial activation of the polymerase, then 15 s at 94 °C for denaturation, one min at 55 °C for annealing, and 45 s at 72 °C for extension repeated 35 times; for final extension, 10 min at 72 °C were used. Duplicated PCR products were then pooled, and PCR amplification success was confirmed using a 1.0% agarose gel. Pooled samples were used for clean-up using E.Z.N.A. Cycle Pure Kit (Omega Bio-Tek, Norcross, GA, USA) following the manufacturer’s instructions and 2.5 μL of the clean product was used for the second PCR step, where each sample was assigned with a unique pair of barcodes (Table S3). The PCR amplification program used was the same as before except 20 cycles were used instead of 35. All samples were pooled into one library and the resulting pool was cleaned with the E.Z.N.A. Cycle Pure Kit. Library concentration was assessed with a Qubit 2.0 fluorometer (ThermoFisher Scientific, Waltham, MA, USA) and then diluted to 10 nM. The library pools were sequenced at the Science for Life Laboratory in Stockholm, using an Illumina MiSeq and 600 cycles, yielding paired-end reads of 300 bp length. Raw data were demultiplexed and quality filtered at the sequencing facility prior to delivery.

### 4.5. Sequence Analysis

The Illumina data were processed using the pipeline proposed by Castro et al. [83] using QIIME2 [89] version 2019.1. Briefly, raw sequence data were imported using the q2-import plugin using the setting for Illumina fastq files, followed by denoising with DADA2 [90] via q2-dada2 denoise-paired, setting truncation at 301 bp for the forward read and 300 for reverse. Taxonomy was assigned with the q2-feature classifier [91] plugin using the UNITE database [92] (version 8.0) at a 97% similarity and dynamic level for fungi and SILVA database [93] (release 132) at a 99% similarity. Amplicon Sequence Variants (ASVs) were aligned with MAFFT [94] and used to infer a phylogenetic tree using FastTree [95], both processes were run in the q2-phylogeny pipeline align-to-tree-mafft-fasttree using default settings.

### 4.6. Statistics

All statistical analyses were performed in R (Version 3.5.3) [96]. Differences in soil mineral content were assessed using Permutational Analysis of Variance (PerMANOVA) with the “adonis2” function from the vegan package [97]. Significant differences were further assessed using the “kruskal” function of the agricolae package [98] to perform the Kruskal–Wallis Rank Sum Test (Response factor ~ Soil). The function provides Fisher’s least significant difference (LSD) as post-hoc analysis and statistical grouping based on Bonferroni correction when significant differences were detected in the Kruskal–Wallis test (α = 0.05). Principal Component Analysis (PCA) was performed on a scaled normalized matrix, using a biplot for visualization plotted with the “autoplot” function from the ggplot2 package [99]. Seedling survival was tested using a Kaplan–Meier curve with the “survfit” function and the proportional hazards regression model with the “coxph” function, both from survival package [100] to assess seedling survival probability and Hazard ratio by species and soil type (Response factor ~ Specie + Soil). Differences in growth and stem water potential were tested using the Kruskal–Wallis test to assess plant species (Response factor ~ Specie) and soil type effects (Response factor ~ Soil) on seedlings growth or water potential. The Kruskal–Wallis test was performed to assess the differences in CFUs in the different soils and Dunn’s test analysis was performed when significant differences were detected.

Sequence analysis outputs were analyzed with the phyloseq package [101]. Prior to any analysis, ASVs with lower than 10 sequences were removed from the dataset, using the “filterfun” function from the genefilter package [102]. Additionally, ASVs with an abundance lower than 0.005% per sample type were also removed. By doing this, 6003 low abundant ASVs were removed from the bacterial dataset and 1358 from the fungal dataset. Finally, samples with a library size lower than 10,000 filtered reads were removed from the dataset. After filtering, species richness was estimated using the “pd” function of the picante package [103] after rarefaction of the samples to 11,619 for the bacterial dataset and 13,136 reads for the fungal dataset, which were the minimum number of reads for a sample in their corresponding dataset. The Shannon diversity index was estimated from raw, non-rarefied samples using the “diversity” function from vegan. Kruskal–Wallis Rank Sum Tests were performed to assess the effect of species (Shannon Index ~ Species) and soil type (Shannon Index ~ Soil) on microbial diversity. Beta diversity was tested using the “ordinate” function from phyloseq package, using Bray–Curtis to build the distance matrix with the rarefied samples, and Principal Coordinate Analysis (PCoA) was used for visualization. Changes in the community composition were tested using PerMANOVA, testing the effect of different soils and sample types. Kruskal–Wallis was used to test any further differences between the different levels. Statistical groups were assigned based on Fisher’s LSD test. Most different taxa of the microbial community were tested using a dissimilarity test [104], using the “simper” function from the vegan package, and using a cumulative contribution up to 70% and permutation *p*-value equal to, or lower than, 0.05 for taxa selection.

## Figures and Tables

**Table 1:** Soils characterization. Numbers represent mean value ± Standard deviation of *n* = 3 soil samples. Superscript lowercase letters represent Fisher’s least significant difference (LSD) when α = 0.05 from Kruskal–Wallis testing differences between soils.

	Native		Foreign	
	Desert	Inland Steppe	Coastal Steppe	Mediterranean
Conductivity (mS/cm)	88.6 ^a^	9.1 ^b^	8.3 ^c^	0.8 ^d^
pH	5.3	8.2	8.0	6.3
Organic matter (%) ^1^	11.2 ± 0.8 ^a^	4.7 ± 0.5 ^b^	0.05 ± 0.004 ^b^	11.4 ± 0.5 ^a^
Field capacity (%)	76.6 ± 9.38 ^a^	74.5 ± 5.44 ^a^	65.4 ± 8.78 ^b^	58 ± 4.74 ^b^
**Macro- and micro-nutrients**				
Cl^−^ (mg/kg) ^2^	22404 ± 89 ^a^	351 ± 1 ^c^	911 ± 17 ^b^	298 ± 1 ^d^
PO_4_^3−^ (mg/kg) ^1^	3936 ± 645 ^a^	3591 ± 739 ^ab^	25 ± 3 ^c^	2653 ± 446 ^bc^
B (mg/kg) ^1^	313 ± 0.3 ^a^	9.8 ± 0.01 ^b^	0.08 ± 0.02 ^d^	1.1 ± 0.004 ^c^
SO_4_^2−^ (mg/kg) ^1^	12436 ± 1520 ^a^	450 ± 29 ^b^	153 ± 2 ^d^	191 ± 13 ^c^
CO^2−^ (mg/kg) ^4^	0.03 ± 0.001 ^ab^	0.4 ± 0.02 ^a^	0.03 ± 0.002 ^b^	0.03 ± 0.0004 ^b^
Ca^2+^ (mg/kg) ^1^	367± 4 ^c^	507 ± 2 ^b^	28 ± 3 ^d^	554 ± 3 ^a^
Na (mg/kg) ^1^	24343 ± 26 ^a^	129 ± 6 ^c^	919 ± 16 ^b^	71 ± 2 ^d^
Li (mg/kg) ^1^	56 ± 1 ^a^	0.01 ± 0.01 ^d^	46 ± 2 ^b^	0.03 ± 0.01 ^c^
Mg (mg/kg) ^1^	117± 11 ^a^	4 ± 0.1 ^d^	17 ± 2 ^c^	59 ± 1 ^b^
K (mg/kg) ^1^	1685 ± 8 ^a^	153 ± 0.4 ^c^	46 ± 2 ^d^	159 ± 1 ^b^
Cu (mg/kg) ^5^	1.6 ± 0.01 ^a^	0.8 ± 0.002 ^c^	1.2 ± 0.1 ^b^	0.4 ± 0.01 ^d^
Mn (mg/kg) ^5^	108 ± 0.2 ^a^	76 ± 0.3 ^b^	13 ± 1 ^d^	63 ± 1 ^c^
Pb (mg/kg) ^3^	1.4 ± 0.1 ^b^	4.4 ± 0.03 ^a^	0.9 ± 0.04 ^c^	0.4 ± 0.1 ^d^
Fe (mg/kg) ^5^	12 ± 0.1 ^b^	1.8 ± 0.02 ^c^	124 ± 1 ^a^	12 ± 0.02 ^b^
Zn (mg/kg) ^5^	4.7 ± 0.1 ^a^	1.5 ± 0.1 ^b^	1.5 ± 0.04 ^b^	1.2 ± 0.2 ^c^

^1^ [47] ^2^ [48] ^3^ [49] ^4^ [50] ^5^ [51] References for methods. See Table S1 for detailed information.

**Table 2:** Bacterial and fungal root-associated community by *Prosopis chilensis* and *P. tamarugo* relative to the ASV abundance in the soil. ASVs with higher abundance in the roots were considered as root associated. Values correspond to the number of ASVs. Value between brackets corresponds to relative proportion in the community, in percentage (%).

	*Prosopis chilensis*			*Prosopis tamarugo*		
	Higher in Root	Equal in Both	Higher in Soil	Higher in Root	Equal in Both	Higher in Soil
**Bacteria**						
Native within	352 (37%)	473 (49%)	131 (14%)	344 (36%)	474 (50%)	138 (14%)
Foreign within	102 (11%)	638 (67%)	216 (22%)	264 (28%)	477 (50%)	215 (22%)
Foreign outside	105 (11%)	684 (72%)	167 (17%)	350 (37%)	463 (48%)	143 (15%)
**Fungi**						
Native within	99 (38%)	116 (44%)	47 (18%)	75 (29%)	140 (53%)	47 (18%)
Foreign within	29 (11%)	163 (62%)	70 (27%)	13 (5%)	178 (68%)	71 (27%)
Foreign outside	40 (16%)	150 (57%)	72 (27%)	39 (15%)	156 (60%)	67 (25%)

## Data Availability

The dataset generated and analyzed in this study is available in the European Nucleotide Archive (ENA; https://www.ebi.ac.uk/ena/browser/home (accessed on 25 April 2022)), under the accession number PRJEB52439. Raw data preprocessing, and the script with the statistical analyses are available online in a GitHub repository (https://github.com/davcastrom/Prosopis_chile_metagen (accessed on 1 March 2022)).

## Supplementary Materials

The following supporting information can be downloaded at: https://www.mdpi.com/article/10.3390/plants11202717/s1, Figure S1: Map of Chile showing *Prosopis* distribution and conservation status; Figure S2: Soil water holding capacity; Figure S3: Common bacteria and fungi; Table S1: Soil characterization methods; Table S2: Primers sequences; Table S3: Indexing combination; Table S4: Soil dissimilarity analysis; Table S5: List of significant bacteria and fungi from dissimilarity test; Table S6: Bacterial community dissimilarity analysis; Table S7: Fungal community dissimilarity analysis.
