# Supplementary material for: Soil Microbiome Influences on Seedling Establishment and Growth of Prosopis chilensis and Prosopis tamarugo from Northern Chile
Source: Plants (Basel). 2022 Oct 14;11(20):2717. doi: 10.3390/plants11202717 (PMC9610084; doi:10.3390/plants11202717)
Supplement: Supplementary file 1 [file Fig_S3.pdf › Supp_material/Fig_S3.pdf]

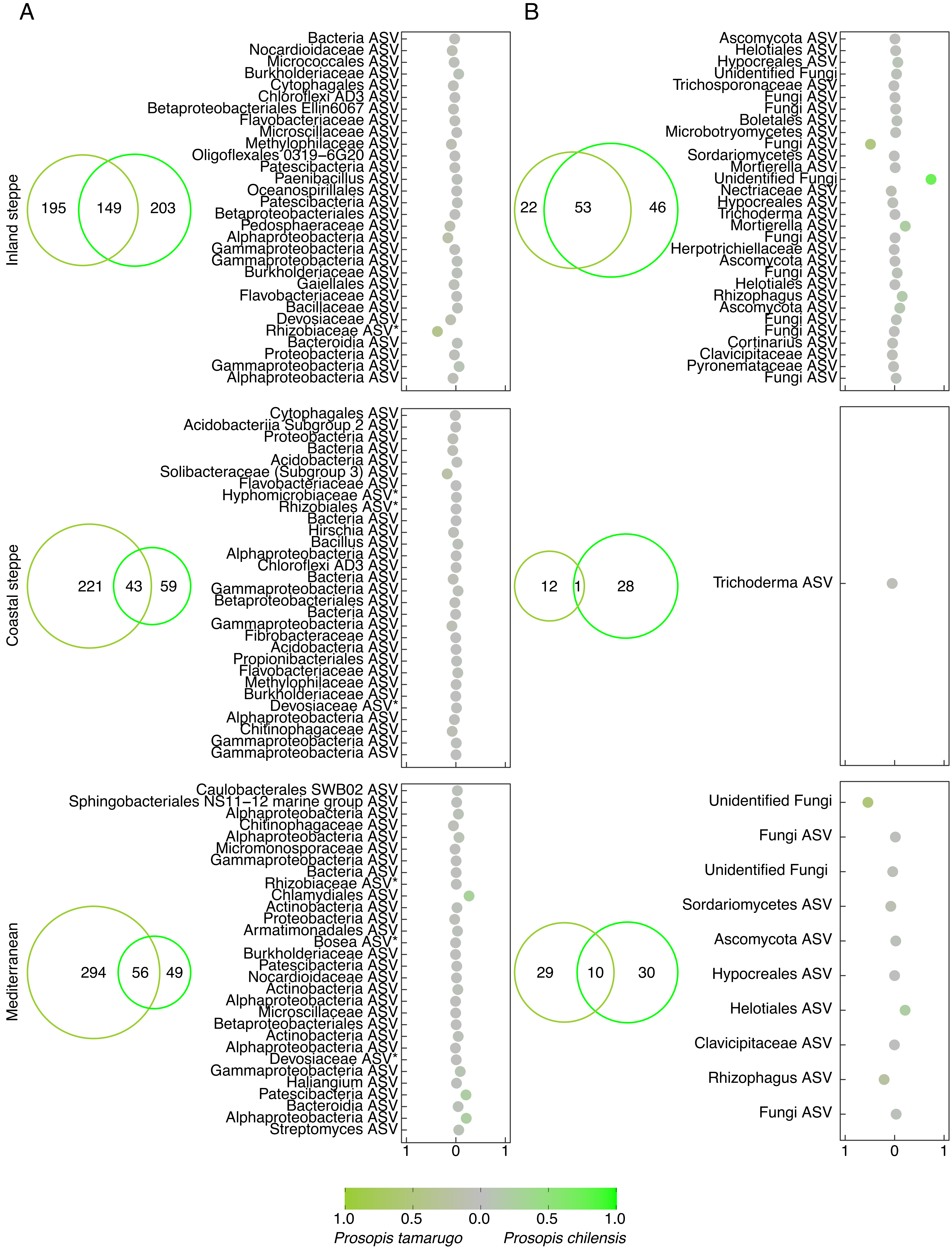


**Fig. S3** Common bacteria (**A**) and fungi (**B**) recruited by *P. chilensis* and *P. tamarugo*. Venn diagrams corresponds to ASVs higher in the roots from Table 2, colored by plant species. Dot plot shows common ASVs for both *Prosopis* species, colored by presence in the roots. Dark green corresponds to ASVs more present in *P. tamarugo* and light green to ASVs more present in *P. chilensis*. An asterisk (*) next to bacterial ASV correspond to *Rhizobiaceae* annotated bacteria.
