# Supplementary material for: Soil Microbiome Influences on Seedling Establishment and Growth of Prosopis chilensis and Prosopis tamarugo from Northern Chile
Source: Plants (Basel). 2022 Oct 14;11(20):2717. doi: 10.3390/plants11202717 (PMC9610084; doi:10.3390/plants11202717)
Supplement: Supplementary file 1 [file plants-11-02717-s001.zip › Supp_material/Fig_S1.pdf]

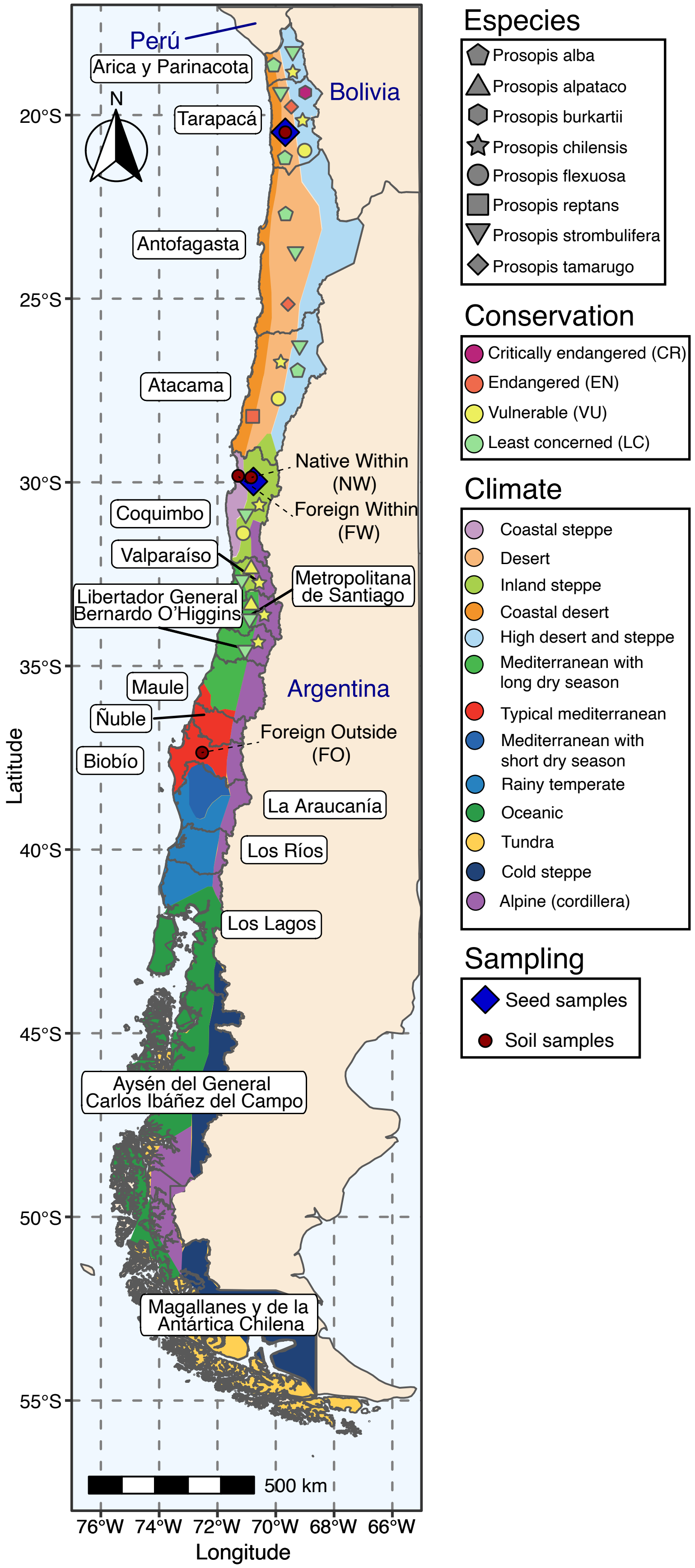

Fig. S1 Map of Chile showing Prosopis species distribution and conservation status. Climate throughout Chile is depicted by different coloring across the regions. Region names in white boxes. Seed sampling locations are shown as a blue diamond and soil sampling locations as red dots. Conservation status taken from http://espe-cies.mma.gob.cl
