# Supplementary material for: Soil Microbiome Influences on Seedling Establishment and Growth of Prosopis chilensis and Prosopis tamarugo from Northern Chile
Source: Plants (Basel). 2022 Oct 14;11(20):2717. doi: 10.3390/plants11202717 (PMC9610084; doi:10.3390/plants11202717)
Supplement: Supplementary file 1 [file plants-11-02717-s001.zip › Supp_material/Fig_S2.pdf]

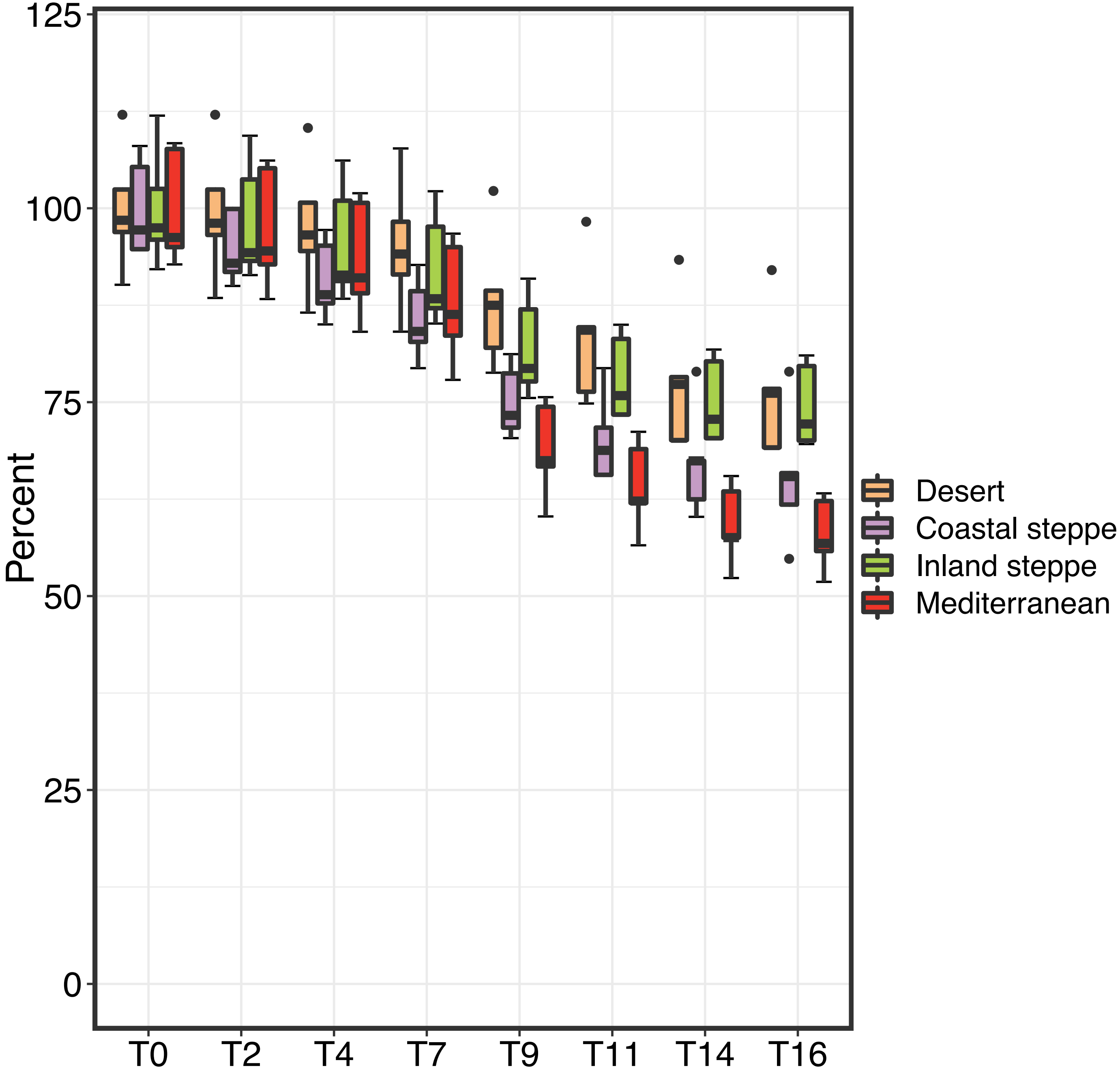

Fig. S2 Soil water holding capacity (in percentage) across 16 days of test. Samples colored by soil type
